# Combating Microbial Infections Using Metal-Based Nanoparticles as Potential Therapeutic Alternatives

**DOI:** 10.3390/antibiotics12050909

**Published:** 2023-05-15

**Authors:** Rajwinder Kaur, Kirandeep Kaur, Mohammad H. Alyami, Damanpreet Kaur Lang, Balraj Saini, Mohammad F. Bayan, Balakumar Chandrasekaran

**Affiliations:** 1 Chitkara College of Pharmacy, Chitkara University, Punjab 140401, India; 2 Department of Clinical Safety and Pharmacovigilance, Soterius India Private Limited, Nehru Place, Delhi 110019, India; 3 Department of Pharmaceutics, College of Pharmacy, Najran University, Najran 66462, Saudi Arabia; 4 Faculty of Pharmacy, Philadelphia University, P.O. Box 1, Amman 19392, Jordan

**Keywords:** metals, nanoparticles, antimicrobial action, microbicidal, biofilm formation inhibitors

## Abstract

The nature of microorganisms and the efficiency of antimicrobials have witnessed a huge co-dependent change in their dynamics over the last few decades. On the other side, metals and metallic compounds have gained popularity owing to their effectiveness against various microbial strains. A structured search of both research and review papers was conducted via different electronic databases, such as PubMed, Bentham, Springer, and Science Direct, among others, for the present review. Along with these, marketed products, patents, and Clinicaltrials.gov were also referred to for our review. Different microbes such as bacteria, fungi, etc., and their diverse species and strains have been reviewed and found to be sensitive to metal-carrying formulations. The products are observed to restrict growth, multiplication, and biofilm formation effectively and adequately. Silver has an apt use in this area of treatment and recovery, and other metals like copper, gold, iron, and gallium have also been observed to generate antimicrobial activity. The present review identified membrane disruption, oxidative stress, and interaction with proteins and enzymes to be the primary microbicidal processes. Elaborating the action, nanoparticles and nanosystems are shown to work in our favor in well excelled and rational ways.

## 1. Introduction

Several kinds of microorganisms lead to the initiation and further development of microbial infections. Such infections primarily and solely manifest in many pathological conditions with variant degrees of severity. Their pathologies precipitate numerous mild symptoms (fever, fatigue, nausea, headache) to serious symptoms (cyanosis, tissue necrosis, lymphadenopathy, respiratory effects) [1,2]. These have also evolved to be one of the major secondary factors in various diseased conditions, while in some, they ultimately cause death [3,4]. Microbial infections and their manifestations interfere at every step of medical methodologies from concluding misleading and erroneous diagnoses to resulting in deleterious surgeries as well as unsuccessful and incomplete treatments [5]. The severity of the condition worsens when there is any kind of additional or major infection. All of this has made antimicrobials a very important and fundamental part of therapeutics and pharmacology [6,7].

The last century has seen excessive use of antibiotics for various kinds of infections. They work by targeting bacterial cell components and altering necessary processes like DNA replication, cell wall synthesis, etc. [8,9,10]. However, they do have certain drawbacks that make them insufficient and problematic in various ways:

- They may affect healthy bacteria present in the body [11,12].
- Monitoring its effectiveness is difficult and challenging [13,14].
- Inconsistency in therapeutic security and the number of associated side-effects [15].

Along with these deficiencies, increasing antibiotic resistance has hinted at the clinical need for newer antimicrobials to tackle microbial growth and biofilm production effectively. Continuous mutations, irrational use of antibiotics, and the production of enzymes that inactivate the bacterial cells have contributed to this increasing resistance to the agents [16,17].

Barring antibiotics, metal compounds were largely in use and practice before the 1920s, after which antibiotics took over [18,19,20]. The potential of metals to conveniently restrict biofilm production make them the best possible alternative in present times [21,22]. Antimicrobial properties of metals have been used since ancient times for disinfecting food and water, managing plant diseases in agriculture, and in medical areas as well [23,24]. Certain metals are necessary for cell functioning and cell membrane formation but their presence in excess amounts can be lethal, whereas specific other metals like non-essential groups, such as mercury, silver, etc., are found to be microbicidal even at very low concentrations [25].

Antimicrobial-metallic agents are now being explored due to their capability to tackle multi-drug resistant bacteria, biofilms, and resistant biofilms, as well as to inhibit important metabolic pathways, plus their suitability with other biocides [26,27,28]. This review compiled and presented the applications of various metal-based nanoparticles as potential antimicrobial agents, including their mechanism of antimicrobial activities. For this work, we have considered a total of 200 papers from electronic databases like PubMed, Bentham, Springer, and Science Direct. We have selected 126 papers covering research and review articles published over the past 10 years, and we have selected keywords such as “metal-based nanoparticles”, “antimicrobial drug-resistance”, and “metal oxides”. Along with these, marketed products, patents, and clinical trials data were also searched in order to write this review article.

## 2. Metals as Antimicrobials

Metals are abundant in the earth’s crust and ecosphere. The Great Oxidation Event (GOE), which took place 2.3–2.4 billion years ago, exposed bacteria to a wide range of metal ions. The earth’s crust contains a variety of oxidized forms of metal compounds as a result of the atmosphere’s rising oxygen level. Enzymes used metals like copper, iron, and zinc for their redox reactions. Metals are necessary for the process of life but are toxic at high intracellular concentrations, and, thus, cells need a homeostasis mechanism to keep the intracellular concentration constant. Zinc and copper share a similar pathogen-killing mechanism in eukaryotes, where oxidative stress is used to destroy the encapsulated bacterium. Metals like gold, silver, and mercury are extremely poisonous to microorganisms at low concentrations [29].

Metals were once utilized as antibacterial agents, but their industrial usage can harm the ecological system, although they do have a medical use. Infections were treated with arsenic, mercury, silver, copper, zinc, and other elements. Antimony and arsenic are employed as fungicides, rodenticides, insecticides, and to treat protozoal illnesses. While zinc salts can be used to treat diarrhea, copper salts are used to make the Bordeaux and Burgundy mixture, which is used to prevent bacterial and fungal problems in plants and to promote animal growth. Burns can be relieved with silver. Organic mercury compounds are utilized to keep eye drops in good condition. Mercury was utilized as a disinfectant and a syphilis infection treatment. In dental restorations, mercury is combined with copper, silver, and tin [30].

### 2.1. Metal-Based Nanoparticles as Antimicrobials

Metallic NPs of sizes ranging from 1 nm to 100 nm can be synthesized by two approaches, i.e., top-down and bottom-up. The top-down approach involves beginning with the material in bulk, which is then broken down into the size of a nanoscopic scale via ball milling or attrition etc. It is an easy method to employ, but increased accommodated impurities and non-uniform sizes of particles limit its use [31,32]. On the other hand, the bottom-up nanofabrication approach includes variant techniques such as the colloidal synthesis, the sol-gel method, the chemical vapor decomposition process, and the atomic layer deposition among others. The process, though time consuming and tedious, has the benefit of uniform-sized and uniform-shaped smaller particles bearing the least number of defects and controlled surface properties [32,33,34].

The use of metal-based nanoparticles as components in the creation of antibacterial agents has been made possible by nanotechnologies. Metal-based nanoparticles (NPs) demonstrate an effective role in locating and eliminating bacteria through a variety of mechanisms, including attraction to the surface of the bacteria, disruption of the cell wall and membrane, and induction of a toxic mechanism mediated by an increase in oxidative stress (e.g., the production of reactive oxygen species (ROS)) [35,36,37,38]. The creation of oxidative stress is a valuable and effective antibacterial method to combat MDR bacteria, given the absence of new antimicrobial medicines with unique mechanisms of action. Therefore, it is important to identify and properly characterize whether NPs might cause oxidative stress in these bacteria [39,40,41]. Metal-based NPs physically interact with bacterial cell surfaces, disrupt their membrane, and, ultimately, restrict the formation of biofilms [42]. The formation of biofilms also leads to the development of resistance against antimicrobial agents, so their hindrance ultimately restricts the modulation of resistant mutants, too [43,44,45]. The shape of metal-based NPs along with their ultra-small, compliantly controllable size, and resultant greater surface area to mass ratio all contribute to the prevention of biofilm formation [46,47,48]. The target microorganisms and their mechanisms of action for a few metal-based nanoparticles are provided in Table 1.

The targeted drug delivery approach of metal-based NPs can be achieved through either active or passive targeting. Active targeting involves the modification of surfaces of metal-based NPs and processes like magnetic targeting, receptor targeting, and other approaches such as temperature-dependent cell death patterned targeting. Passive targeting, meanwhile, is generally accomplished by improving permeation and enhanced retention at the site of infection [55].

Within a single metal-based NP, multiple drugs can be accommodated to bring an elaborative action. Different drugs that have different kinds of mechanisms will exert a joint action and will subsequently result in higher efficiency. The microbe being either resistant or a multi-drug resistant mutant will probably be successfully affected via one or the other of the variant processes [56,57].

### 2.2. Mechanisms Involved in Antimicrobial Activity of Metal and Metal-Based NPs

**Figure 1:**
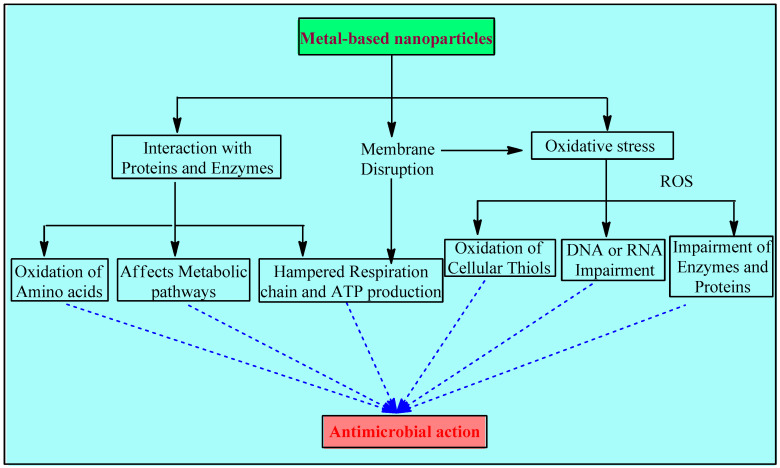
Mechanisms involved in the antimicrobial activity of metals and metal-based NPs.

Metal and metal NPs interfere with bacteria’s hemostasis in 3 major ways: disruption of the membrane, oxidative stress, and interaction with proteins and enzymes (Figure 1).

#### 2.2.1. Disruption of the Membrane

Associating metal nanoparticles with conducting polymers that have positive charges on their surfaces is essential for stabilizing solutions with high nanoparticle concentrations. According to the bacterial eradication mechanism, cell death is caused by electrostatic contact between the negatively charged bacteria (electronegative groups of the polysaccharides in the membrane) and the positively charged compound, such as metal and metal oxide nanoparticles with a variety of forms, roughness, and positive zeta potentials. Positively charged NPs interact with electro-negatively charged bacterial membranes, directly causing bactericidal toxicity in the membrane and targeting cell integrity The surface charge is an important factor for the antibacterial activity of the metal-based NPs. Positively charged NPs are more effective than neutral or negatively charged ones. As reported in [45], NPs with a positive charge display higher toxicity due to their electrostatic interaction with the negative charge of the bacterial cell wall. A comparative study showed that magnetic NPs with a positive charge (NP+) efficiently attracted over 90% of E. coli, while negatively charged magnetic NPs (NP−) did not show any affinities. These results suggest that NPs+ have good potential to capture bacteria via electrostatic attraction. In another study, the antibacterial efficiency of three different AgNPs that were positively, negatively, and neutrally charged were compared, and it was found that positively charged NPs showed the highest bactericidal activity, while the negative ones showed the lowest [58,59,60].

Electron microscopic studies over *S. aureus* and *E. coli* also indicate the compromised cytoplasmic membrane’s integrity due to toxic doses of certain metals such as aluminum and silver. A few agents, especially silver, impede the electron transport chain [ETC] [61,62]. Cell wall synthesis is also interrupted due to the interaction between the sulfur-containing constituents of the membrane and the silver nanomaterials. Apart from this, toxic doses of copper and cadmium have also been thought to cause lipid peroxidation [60,63,64].

All such disruptions lead to oxidative stress and, subsequently, to further damage, such as interrupted energy homeostasis, impeded respiration, and, ultimately, cell death [65].

#### 2.2.2. Oxidative Stress

The metal-based NP system provides the benefit of controlling the particle specifications, including its shape, size, and even the charge on the surface, and it also provides the option to manage the release of metal ions [66]. The mode of their action is generally linked with the disruption of the membrane initially and the generation of ROS in large amounts gradually. The driving force of their action is associated with the controlled release of metal ions, while some research suggests that this release is through the leaching of metal complexes with amino acids of bacteria [67]. Metal ions in solution, ROS, and ROS-mediated machinery may all play a role in the toxicity of metal oxide nanoparticles. The potential of CuO NPs for toxicity may be related to problems with DNA synthesis and repair as well as an increase in the production of reactive oxygen species [68,69]. Higher antimicrobial activity of smaller metallic NPs is reported compared to larger NPs, and this is due to their larger surface area to volume ratio, which increases the production of ROS [70].

The introduction of metal-based NPs initiates intracellular ROS production, which has been confirmed by the electron paramagnetic resonance technique [41]. Such oxidation reactions are catalyzed by numerous metal ions, such as copper, iron, chromium, arsenic, etc., which upregulate genes and also cause other damage [71]. Iron brings in auto-oxidation through aerobic respiration, which leads to the production of oxygen radicals and hydrogen peroxide [72].

Moreover, the consumption of cellular anti-oxidants may also begin during this redox cycling phase of metals. Oxidative imbalance causes oxidation of cellular thiols as well, which, in turn, develops covalent bonds with sulfur. Ultimately, this results in the formation of protein disulfides and the reduction of anti-oxidant sources [70,73,74,75]. Oxidative stress led by ROS impairs the DNA or RNA, attacks enzymes and proteins, and thereby damages macromolecules [76].

#### 2.2.3. Interaction with Proteins and Enzymes

Metallic NPs initiate an antimicrobial response by binding to cytosolic proteins. The interaction of these NPs with enzymes and DNA hampers the whole homeostasis. Since metabolic pathways are affected, the respiratory chain, ATP production, and replication processes are impaired and ultimately inhibited [77,78].

### 2.3. Bio-Medical Antimicrobial Applications of Metal-Based NPs

Multi-drug resistant organisms are often resistant to commonly used antibiotics. The lack of and the great need for effective antimicrobial agents have led to the development of novel strategies to address this growing public health issue. A growing number of drug-resistant mutants and the inability to cure infective conditions completely has fueled the production of NPs, and this has now found several applications:

Dental products—Microbes tend to settle on teeth, leading to the development of plaque, thereby increasing the chance of mouth infections. NPs not only potently restrict the growth of bacteria but nano-crystallization also improves their performance and inhibits the formation of biofilms as well [79].

Coating of implantable devices—Coating of implantable devices like heart valves prevents adhesion and further growth of bacteria like *E. coli*, reducing the risk of inflammation and infections [80,81]. Devices that are commonly prone to the colonization of bacteria, such as dental implants, catheters, etc., are generally subjected to NP coatings [56,62].

Wound dressings—Several microbes like *Streptococcus*, *E. coli*, and *Staphylococcus*, among others, can cause wound infections, inflammation, and other complications that can be precipitated. To significantly prevent this, broad-spectrum antimicrobial NPs are an option [82,83].

Other than these, NPs can often be used along with bone cement and also in certain drug delivery systems [84]. It has been stated that we are on the verge of the ‘post-antibiotic era’ by the Centre for Disease Control and Prevention (CDC). The prediction of more deaths due to antimicrobial resistance than cancers by 2050 has also been made by many, and all of these predictions and statistics have led to the desire for newer molecule options and drug delivery methods as well as post-numerous research and studies, and the use of metal-based nanoparticles seems to be an effective lead here [85]. Indeed, the latter have been spotted to target resistance and biofilms via different mechanisms and pathways, depending on the metal and its potential.

## 3. Metal-Based Nanoparticles Mediated Antimicrobial Effects

Metal is precipitated by bacteria as protein aggregates, sulfides, oxides, or elemental crystals. The formed precipitates then interact with the membrane and move into the cell, initiating antimicrobial effects via different mechanisms and gradually, terminating the planktonic growth.

### 3.1. Silver Ions and Silver Nanoparticles

Silver [Ag] has been in use for water purification, preservation, anti-fouling, anti-fungal, antiseptic, and antibiotic effects for many ages now, and it has the potential to mitigate or even solve considerable medical burdens in our societies [86]. Indeed, silver-impregnated bandages, devices, and other equipment have shown effective antimicrobial action, and this effect is commonly witnessed in practice [87].

Silver requires the formation of silver ions to develop bactericidal action. The release of these ions is through retarded oxidation, which is a slow process that gradually results in lower efficient silver levels. Thus, since the 1st century BC, silver salts have been used [88]. Silver ions tend to interact with the membrane, and later pursue microbicidal action. Silver NPs are known to be more effective, and they strongly anchor to the membrane and keep on releasing biochemically active silver ions in small amounts. As the ions are released, pit formation is initiated by the metallic silver in the cell wall of bacteria. Pit formation gradually increases the permeation of the membrane, which impedes necessary transport processes. Slowly, lysis is precipitated, since vital membrane proteins and cellular components begin to leak out of the pits [89,90,91].

Along with this, silver ions bind to reduced thiol groups and interfere with iron-sulfur [Fe-S] clusters, which are vital for biochemical processes such as electron transfer and respiration [92,93].

Electron donor groups of proteins and silver ions share an extremely firm and powerful electrostatic interaction, which indirectly gives access to Ag ions to interfere with the proteins [94]. Metal chelation sites inside the enzymes are occupied by Ag ions, causing enzyme inactivation [95]. This not only hampers necessary cellular processes but also impairs the cell transport system and cell wall integrity. In the later stages, the cell undergoes necrosis led by silver ion-induced DNA condensation and the lack of replication capacity [96].

Apart from bacteria, silver NPs are seen to be active even against certain viruses––namely, murine norovirus and HIV-1 among others. They tend to inactivate certain bacteriophages and restrict the virus’ capability to infect the host cells by targeting disulfide groups present in surface proteins [97,98].

One of the first found and used silver salt-silver nitrates has high solubility, and consequently leads to a higher concentration of silver in the cell, while silver sulfides and silver halides are sparingly soluble agents that release silver slowly. Another salt that has been in use for decades for its antibiotic effects is silver sulfadiazine. It is a sulfonamide as well, bringing out a more elaborative action. The use of silver nanoparticles in combination with fluconazole and with other clinically approved marketed drugs showed a better resistance to fungal infection [99,100].

Ag^+^ ion, the most stable ion of silver, bears a moderate oxidizing ability, while the higher oxidation states’ species- Ag^2+^ and Ag^3+^ are extremely strong oxidants but have not been explored as much, owing to their instability in solution. A potential approach to the instability of the higher oxidation states can be Silver Oxysalts [Ag(Ag_3_O_4_)_2_X]. The most stable of all oxysalts of silver is the nitrate-coordinated moiety silver oxynitrate [101].

NPs with domains of silver and covering shells of gold were tested over separate strains and in combination strains of *S. aureus*, *P. aeruginosa*, *E. coli*, and *Enterococcus faecalis*. The combination NPs were seen to produce dose-dependent effects while inhibiting biofilm formation. They also permitted specific and faster leaching and interaction of silver ions into cellular components. The damaging process was observed to be immediate and quicker at every stage of the initiation of cellular damage, disintegration, and lysis, owing to the increased affinity of silver to molecules precipitating the inactivation of enzymes and certain proteins, which hindered ATP production [102].

#### Silver Oxynitrate [Ag(Ag_3_O_4_)_2_NO_3_ or Ag_7_NO_11_]

Silver oxynitrate is a silver oxysalt, containing stable higher oxidation state ion species of silver, and it is now being explored because of excellent effects observed in various types of research. It is not only effective against antibiotic-resistant and multi-drug-resistant mutants but has also been seen to target biofilms. Biofilms are one of the prominent reasons for resistance development and they also slow the healing process, gradually creating a chronic non-healing infection [103].

Doherty et al. reported that the new silver dressing promises to fight wound infection, although its anti-biofilm effectiveness and benefits on healing apart from infection are sometimes ambiguous. The dressings exhibited a distinct impact on the healing of biofilm-infected and uninfected wounds, with Ag Oxysalts dressings having a more favorable effect than the control therapy and the other silver dressings on re-epithelialization, wound size, and inflammation. The study was carried out by using in vitro and in vivo *S. sureus* and *P. aeruginosa* biofilm models [104].

This involved a comparison between Ag_7_NO_11_, AgNO_3,_ and CuSO_4_ to tackle dual-species planktonic biofilm population, where MIC of silver oxynitrate was found to be remarkably lower than that of CuSO_4_ and AgNO [105]. A list of a few antimicrobial marketed products containing silver is provided in Table 2. Apart from the listed ones, certain other dressings and medical products exist using silver metal for its antimicrobial potential, such as Mepilex^®^ Ag. This is a silicone foam-type dressing, capable of action within 30 min, providing an effect on wounds, burns, and certain ulcers [106].

### 3.2. Copper and Copper Nanoparticles

Copper was the first metal element to have been used by mankind, and it was used for various reasons. It is also mentioned in one of the oldest books ever written, *Smith Papyrus*. Egyptian, Roman, and Greek medical literature have noted its benefit in treating ear infections, burns, and intestinal worm-caused infections and headaches, as well as its use for sterilizing water and wounds. The nineteenth and twentieth centuries witnessed the increased use of copper in treating ailments such as syphilis, tuberculosis, anemia, lupus, facial neuralgia, eczema, adenitis, and gastric ulcers [111].

**Figure 2:**
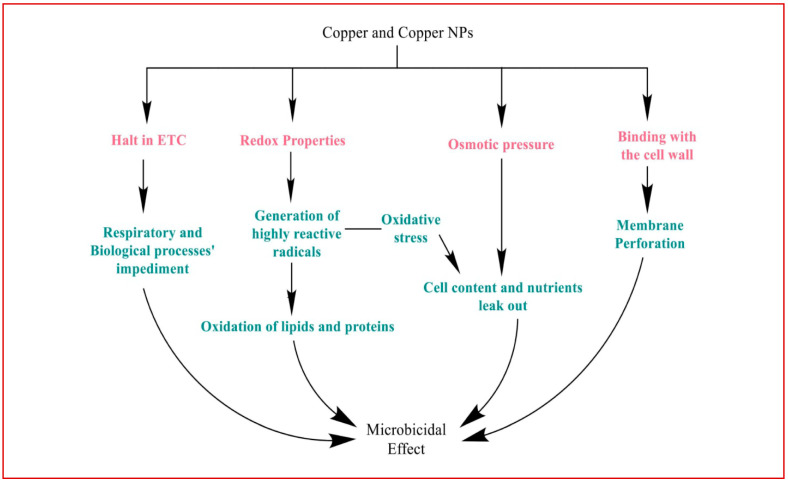
Various mechanisms mediated the antimicrobial action of copper on bacteria and its homeostasis.

Copper binds to the cell wall of bacteria, directly hampering membrane proteins and initiating membrane perforation. Ions, here, begin interacting with sulfur and phosphorus-containing moieties such as certain proteins, enzymes, and DNA (Figure 2) [112].

Cellular damage by copper generally begins because of its redox properties. A fenton-type reaction involving copper ions and hydrogen peroxide leads to the generation of highly reactive hydroxyl radicals. These radicals, in turn, target lipids and proteins, and oxidize them. Subsequently, electron transport is interrupted along with the peroxidation of lipids, impeding biological and respiratory processes in the end [113,114,115].

Copper damages the membrane of the cell through disturbed osmotic pressure and oxidative stress, causing cell contents and nutrients to leak out, and precipitating cell death. Another known mechanism of damage is through displacing iron from clusters of iron and sulfur. The competition of copper ions with other metal ions such as zinc on proteins for the binding sites, as well as inappropriate binding, leads to the loss of function of proteins, which gradually breaks apart the protein into many non-functional segments [113,116].

Copper is generally seen to be effective against both gram-negative and gram-positive bacteria. Abbas et al. reported that copper nanoparticles have antibacterial activity, where copper nanoparticles have an effect on gram-negative microorganisms by showing an impact on the cell film of microscopic organisms, i.e., *S. aureus* and *E. coli* [117]. Another study carried out by Sharma et al. shows that colloidal copper nanoparticles have an antibacterial activity against gram-negative bacteria (*E. coli* and *Proteus vulgaris*) under culture conditions. By using the chemical reduction method, three sets of Cu nanoparticles were synthesized and antibacterial activity was calculated by minimum inhibitory concentration and minimum bactericidal concentration, reactive oxygen species, and cytoplasmic leakage assays. Copper nanoparticles exhibit good antibacterial activity [118].

As is the case with silver, copper also gained importance in the past due to its effectiveness against a broad spectrum of species and multi-drug resistant species, and it also tends to be a cheaper and more easily available than silver and gold products. The synthesis and fabrication of these NPs has given us products with better physical and chemical properties, owing to their potential to undergo oxidation and form copper oxide NPs [114].

### 3.3. Gold and Gold Nanoparticles

Metal gold [Au] carries properties for cytotoxic and genotoxic effects, supported by its characteristics of being inert and highly stable, though it requires a high concentration to initiate the antimicrobial effect. The potential is intensified when its size is reduced to formulate a nanomaterial. Similar to the enzyme glucose oxidase-catalyzed hydrogen peroxide generation, gold NPs increase ROS production. They tend to upregulate the oxidative enzymes and, on the other side, downregulate reductive enzymes, thus creating a state of metabolic imbalance. The generated oxidative imbalance gradually impedes cell functioning [119,120].

Along with this, these nanoclusters bring about irreversible damage to the membrane by downregulating genes associated with the proteins that bind to the surface of the cell wall and subsequently upregulate membrane stability and integrity. Theories also suggest that Au NPs impede vital processes such as transcription and translation, and that they promote their collapse [121].

The antifungal effect of gold and its NPs was seen to be dependent on size and concentration. A study by Salehi et al. reported excellent antifungal action against 58 Candida spp., for example. For the experiment, the polymerase chain reaction fragment length polymorphism and HWP1 gene amplification approach methods were employed to identify Candida spp. The synthesized CAS-AuNPs were characterized using transmission electron microscopy (TEM) and the Zetasizer system, and they were evaluated by SEM. Results were found to be beneficial with a low MIC value of ≥4 µg/mL for CAS. A TEM result was also found with an average size of 20 nm, and the Zeta potential of CAS-AuNPs was found to be −38.2 mV. The statistical analyses showed that CAS-AuNPs reduce the min. IC_50_ against *C. albicans* with a *p* value of 0.0005 and non-albicans Candida with a value of *p* < 0.0001. SEM confirms the effect of AuNPs on the *C. globrata* cell wall structure by the formation of pores. Conclusively, thereof, the better antifungal action of CAS-Au NPs has observed the encapsulation of antifungal drugs in combination with NPs [122].

### 3.4. Iron and Iron-Based Nanoparticles

The use of iron for conditions like iron deficiency is well known, but the need for novel therapeutic products has led to the identification of various other indications for administering iron and iron NPs, which have worked wonders for patients. They have been found to have the potential to be anti-cancer, immunosuppressive, anticonvulsant, anti-inflammatory, antibiotic, and antifungal agents [123].

The effectiveness of iron as an antimicrobial has been observed to be tremendously good, generating toxicity via several mechanisms involving membrane depolarization, hampering membrane and cell integrity, and mediating damage through oxidative stress that, in turn, affects the homeostasis of bacteria [124].

Iron tends to enter the membrane through diffusion or endocytosis, and it facilitates the production of ROS. The generated oxidative stress and ROS lead to the direct interference of electron transport at the stage of oxidation of bacterial NAD, which is the essential co-factor involved in various cellular processes. ETC is stimulated in a way that generates superoxide ions, which subsequently target Fe-S clusters. This damage affects all necessary biochemical pathways, impeding survival [125,126].

Superparamagnetic iron oxide, through the Fenton reaction, not only accelerates the production of ROS and oxidative stress but also targets protein, lipids, and DNA. Ultimately, lipid peroxidation terminates into ferroptosis [127].

When iron enters the cell via endocytosis, the NPs accumulate in lysosomes and initiate lysosomal destabilization. From this cell, destruction is followed as growth is arrested, leading to gradual autophagy and cell death. Along with this, the NPs potentiate biocidal activity by serving as antibiotic-carrier nanosystems. This kind of physical mixture shows rapid and elaborative activity [123].

### 3.5. Gallium and Gallium Nanoparticles

Gallium [Ga], though bearing no natural function of its own, takes advantage of bacteria’s disability to differentiate between iron and gallium ions. Iron is required by bacteria for most of its processes of growth, maintaining metabolic balance, and replication. The reduction of ferric ions to ferrous ions helps the bacterial enzymes to avoid the state of increased ROS. Gallium uses the uptake mechanism of ferric ions to make its entry into the cell. Gallium ions, though mimicking iron, cannot be reduced as ferric ions, thus terminating redox reactions and disturbing the whole hemostasis. This hampers normal electron transfer, and it inhibits the generation of ATP, thereby disrupting the respiratory process. The halt in redox reactions impairs DNA synthesis and the ability to replicate, causing cell death [77].

Gallium can be further grafted, along with other metals, for variant applications. It can be implied in some forms like Ga-mesoporphyrin, Ga-hematoporphyrin, Ga-octaethylporphyrin, and Ga-protoporphyrin, etc. [128]. A coordination complex between maltol and gallium–gallium maltolate is demonstrated to be an anti-biofilm formation agent, which terminates the formation of biofilms by decreasing bacterial colony-forming units significantly, and, along with this, also reduces pain and inflammation at low doses [129].

Apart from bacteria, gallium also impedes and gradually halts HIV growth. Ga NPs tend to inhibit the release of Interleukins- IL-6 and Il-8, which are essential in propagating HIV. Gallium possesses the capability of inhibiting both bacterial and viral co-infection of HIV, and *M. tuberculosis* is the first reported formulation to bring about such an effect with great success [130].

### 3.6. Patent Products and Clinical Status of Metals as Antimicrobials

Several metals and metal ions, or their combinations, are in trials. Silver, extensively used for antimicrobial purposes, has more than 100 recruited trials in process. One of the interventional-randomized-single blind trials, initiated in 2018, comparing Silver NPs to an approved antimicrobial topical gel for fungal infection, expects rational, positive results by 2021 [131]. Another much-awaited result is for copper’s activity. This trial aims to appraise the efficacy of copper oxide-bearing dressing for wounds, such as for cases of diabetic foot ulcers and pressure ulcers [132]. Studies on other metals are also in trials for proper and safe application, but they still need to be concluded. Some patents relating to such combinations have been approved and issued claiming effectiveness, and a few of these are listed and elaborated in Table 3.

## 4. Conclusions and Future Prospective

It is becoming increasingly difficult to treat patients and combat infectious diseases in an era of rising MDR, in which bacteria are developing resistance to many different antibiotics and leading to major morbidity and mortality consequences. Metal alternatives for addressing the microbe-mediated infectious burden have been supported by numerous reported investigations and ideas. A better knowledge of microbial metal toxicity heralds a new age for the rational design of metal-based antimicrobial therapies. Nanotechnology might be useful for treating bacterial infections in particular. Metallic NPs are an effective substitute for antibiotics, and show great promise in addressing the issue of the emergence of bacterial MDR. The applications of metallic NPs, whether in antibacterial vaccines to prevent bacterial infections, in antibiotic delivery systems to treat disease, in bacterial detection systems to produce microbial diagnostics, or in antibacterial coatings for implantable devices and medicinal materials to prevent infection and promote wound healing, are just a few of these applications. Bimetallic NPs have also shown additive and elaborative action in addition to metals and their salt oxides.

These molecules have grown in popularity as suitable, apt, and efficient candidate options to formulate into conventional therapies for patients taking proper knowledge, rational use, and suitable implications for technology, especially nanotechnology, on account of their antimicrobial effects. Recent technological advancements include the use of siderophores, antibacterial metal nanoparticles, and abiotic metal surfaces and coatings. Metal-based antimicrobial therapies have a lot of potential as antibiotic substitutes and can be considered as the lead for the development of safer, non-resistant antimicrobials. Further and extensive studies are required to explore and improve the toxicity limits for their usage, though.

## Figures and Tables

**Table 1:** Antimicrobial activity mechanisms of different metal-based NPs.

S. No.	Metal-Based Nanoparticles	Microorganism	Mechanism of Action	Reference
1.	Smaller silica nanoparticles	*E. coli* bacteria	Cell wall rupturing	[49]
2.	AgNPs	*K. pneumoniae*	Damage to bacterial cell wall Reactive Oxygen Species (ROS) generation	[50]
3.	CuNPs	*F. oxysporum*	Structural and functional changes in fungi cell, affects DNA and its replication, and protein synthesis	[51]
4.	AuNPs	*B. subtilis*	Bacterial membrane damage	[52]
5.	Iron nanoparticles	*P. aeruginosa*, *E. coli*, *S. aureus* and *B. subtilis*	Bacterial cell membrane rupture ultimately led to bacteria death	[53]
6.	Gallium based nanoparticles	*P. aeruginosa*	ROS-mediated bacterial cell wall damage	[54]

**Table 2:** List of select antimicrobial-marketed products containing silver.

Marketed Products	Composition	Indications	Brand Name and Manufacturer (City, Country)	Reference
Silverex Heal Gel	Gel: Silver nitrate 0.2% Cream: silver sulphadiazine 1% *w*/*w*, chlorhexidine gluconate 0.2% *w*/*w* Topical powder: silver sulphadiazine 1% *w*/*w*, chlorhexidine hydrochloride 0.5% *w*/*w*	Burns, wound sepsis, skin lesions, skin injuries, skin wounds, Gingivitis	Silverex ionic & Sun Pharma (East Mumbai, India) Silverex SSD & Sun Pharmaceutical Industries Ltd. (Mumbai, India)	[107]
Silvadene	Every gram of Cream 1% contains 10 mg of micronized silver sulfadiazine. Composition of vehicle—white petrolatum, stearyl alcohol, isopropyl myristate, sorbitanmonooleate, polyoxyl 40 stearate, propylene glycol, and water, with methylparaben 0.3% as a preservative	As an adjuvant, in wound sepsis prevention, and treatment and burns (secondary and third degree)	Silvadene & King Pharmaceuticals, Inc. (Bristol, England)	[108]
Megaheal gel	Propylene Glycol 4.96% *w*/*w*, carbomer 0.76% *w*/*w*, Silver colloid 32 ppm, triethanolamine 0.32% *w*/*w*	Bacterial infections	Megaheal & Aristo Pharmaceuticals Pvt. Ltd. (Baddi, India)	[109]
Silvel	Nanocrystalline Silver	Ulcers, carbuncles, abscesses, first and second-degree burn wounds, surgical wounds (active against Pseudomonas, Methicillin-resistant Staphylococcus aureus, and Vancomycin Resistant Enterococcus, and claims to be effective for about 5 days of use)	Silvel & Datt Mediproducts Limited (DMPL) (Una, India)	[110]

**Table 3:** List of patents of formulations containing different metals or metal ions registered for their antimicrobial action.

Patent Product	About the Product	Activity	Reference
Antibacterial and Painless needles	Silver NPs coated-medical/surgical needles bearing painless and antibacterial properties	Prevention of infections	[133]
Silver Hydrosol	Silver suspended aqueous gel	Dental infection risk minimization	[134]
Modified Gold nanoparticles	Au NP modified by amino pyrimidine	Broad-spectrum antibiotic effect	[135]
Formulation of Copper and Quinone	The antimicrobial formulation of a copper salt along with quinine for topical use	Increased antimicrobial effect	[136]
Colloidal silver composition constituting silver and water.	Colorless composition with a particle size of about 5–40 ppm with exterior part constituting ionic silver oxide and interior of elemental silver	Antimicrobial activity	[137]
Gallium containing composition	Composition for impregnating or coating devices to halt formation and growth of biofilms	Biofilm growth inhibition	[138]
Antimicrobial coating of medical implants.	The coating of medical implant contains an antimicrobial agent including metal or/and metal ions like silver, zinc, copper, or their combinations, and a bioactive material	Effective antimicrobial effect	[139]

## Data Availability

No new data were created or analyzed in this study. Data sharing is not applicable to this article.
